# Laccases and Tyrosinases in Organic Synthesis

**DOI:** 10.3390/ijms23073462

**Published:** 2022-03-22

**Authors:** Ludmila Martínková, Barbora Křístková, Vladimír Křen

**Affiliations:** 1 Institute of Microbiology of the Czech Academy of Sciences, Vídeňská 1083, CZ-142 20 Prague, Czech Republic; barbora.kristkova@biomed.cas.cz (B.K.); kren@biomed.cas.cz (V.K.); 2 Faculty of Food and Biochemical Technology, University of Chemistry and Technology, Technická 5, CZ-166 28 Prague, Czech Republic

**Keywords:** laccase, tyrosinase, oxidation, homocoupling, heterocoupling, dimer, oligomer, catechol, bioactive compound, organic synthesis

## Abstract

Laccases (Lac) and tyrosinases (TYR) are mild oxidants with a great potential in research and industry. In this work, we review recent advances in their use in organic synthesis. We summarize recent examples of Lac-catalyzed oxidation, homocoupling and heterocoupling, and TYR-catalyzed *ortho*-hydroxylation of phenols. We highlight the combination of Lac and TYR with other enzymes or chemical catalysts. We also point out the biological and pharmaceutical potential of the products, such as dimers of piceid, lignols, isorhamnetin, rutin, caffeic acid, 4-hydroxychalcones, thiols, hybrid antibiotics, benzimidazoles, benzothiazoles, pyrimidine derivatives, hydroxytyrosols, alkylcatechols, halocatechols, or dihydrocaffeoyl esters, etc. These products include radical scavengers; antibacterial, antiviral, and antitumor compounds; and building blocks for bioactive compounds and drugs. We summarize the available enzyme sources and discuss the scalability of their use in organic synthesis. In conclusion, we assume that the intensive use of laccases and tyrosinases in organic synthesis will yield new bioactive compounds and, in the long-term, reduce the environmental impact of industrial organic chemistry.

## 1. Introduction

**Figure 1:**
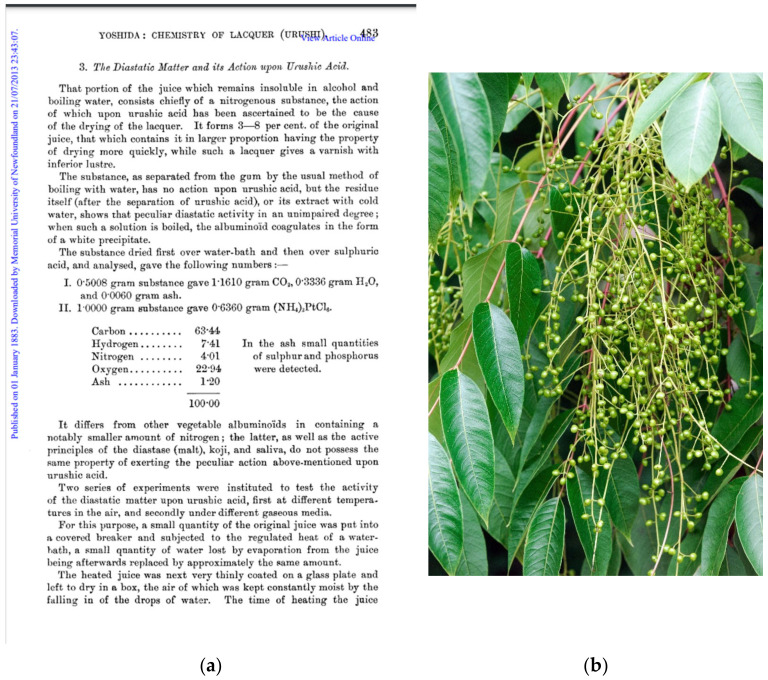
(**a**) The first report of a “diastatic matter”, i.e., laccase, in the “urushi” sap [13], with permission; here: page 483; (**b**) the sap producing tree *Toxicodendron vernicifluum* (iStock.com/Falombini); with permission.

Laccase (Lac; EC 1.10.3.2)—the “blue enzyme for green chemistry” [1] was first described in 1883 as a “diastatic matter” (Figure 1a) from the sap of the tree *Toxicodendron* *vernicifluum* (former name *Rhus verniciflua*; “Chinese lacquer tree”; Figure 1b). The main constituents of the sap are water, urushiol (a mixture of substituted benzene-1,2-diols—catechols), and polysaccharides. Laccase constitutes approximately 1% of the sap and catalyzes the polymerization of urushiol [2]. This sap has been used as varnish (“urushi”) in the traditional manufacture of highly regarded “urushi” goods [2,3,4]. Annual production of the sap was nearly four thousand tons in 2014 [2], but (semi)synthetic alternatives are being sought.

Later, the sources of Lacs were greatly expanded. Lac activities were found in other plants (poplar, sycamore maple, tulip tree, tobacco, maize, rice, oilseed rape, etc.) and in the two largest phyla of fungi (Basidiomycota, Ascomycota). A number of Lacs were also reported in the bacterial genera *Azospirillum*, *Bacillus*, *Streptomyces*, *Escherichia*, *Pseudomonas*, *Thermus*, *Sinorhizobium*, *Oscillatoria*, *Haloferax*, etc., and in various insects [5]. Lacs from different sources differ in their redox potential, which is higher in fungal Lacs (470–810 mV) than in bacterial Lacs or the already mentioned enzyme from the “lacquer tree” (approximately 400 mV) [6].

Lac is a copper protein with four copper atoms in its active site [1]. The copper atoms are classified as T1, T2, T3α, and T3β [7], and have different coordination environments [1,7]. The absorption of the T1 site at 605 nm (in the Lac from *Trametes versicolor*) is the cause of the blue color of Lac [8]. However, some laccases with an altered catalytic site structure may appear “yellow“ or “white“ [8,9,10]. We refer to previous studies [1,7,8,11,12] for an explanation of the catalytic mechanism and the structure-activity relationships in Lacs.

Lac is widely recognized as a mild and environmentally friendly oxidation catalyst with atmospheric oxygen as a reactant and water as a byproduct. The variety of products formed by the use of Lacs is broad. The radicals formed during the reaction yield various products by coupling. In addition, mediators broaden the range of substrates. They allow alleviation of the limitations caused by the redox potential of Lacs and extend their use to non-phenol compounds. 2,2′-Azino-bis(3-ethylbenzothiazoline-6-sulfonic acid) (ABTS) or 2,2,6,6-tetramethylpiperidine-1-oxyl (TEMPO) are widely used in Lac-mediator systems. For example, ABTS has improved the removal of both phenol and non-phenol synthetic dyes (bromophenol blue, xylene cyanol, Coommassie^®^ Brilliant Blue R-250) by an immobilized Lac [14]. ABTS or a nature-derived mediator, ferulic acid, have been useful for the remediation of soil contaminated with polyaromatic hydrocarbons [15]. ABTS was used together with carbon nanotubes to mediate the oxidation of anthracene to the corresponding quinone. This allowed the construction of a new anthracene sensor with high sensitivity [16].

Thus, Lacs can be used in a variety of industries (paper, textile, food), materials engineering, bioremediation, biofuel cells, or biocatalysis. However, most of the proposed applications are still waiting to be used on a larger scale. In the last decade, the properties and uses of Lacs have been summarized in comprehensive reviews [17,18,19] and reviews focusing on subtopics such as applications of fungal Lacs [20], evolution and in vivo functions [5], biodegradation [21,22,23,24], biosensors [25], polymer synthesis [26] and grafting [27], biorefineries [28], food industry [29], wood composite production [30], immobilization [26,31,32,33,34,35,36], and biocatalysis in ionic liquids [37] or Lac mimics [38]. For background knowledge on Lac (structure, Lac-mediator reactions, applications) we refer to the review by Riva [1], and for an overview of laccases, their origin, and properties we refer to the review by Baldrian [39].

The common feature of tyrosinase (TYR; EC 1.14.18.1) and Lac is the involvement of copper in their catalytic cycles. However, TYR contains two copper atoms coordinated by His residues. The oxidation state of copper changes between Cu^+^ and Cu^2+^ within the catalytic cycle as in Lac. The reaction mechanism of TYR has been discussed previously [40,41].

The range of TYR applications is smaller compared to Lacs, as their substrate scope is not as broad. However, TYRs offer additional possibilities: In organic chemistry, they have been used to catalyze *ortho*-hydroxylations. They can also be useful in the modification of biopolymers [42] and biodegradation [21]. Most recent reviews on TYRs have focused on TYR inhibitors [43,44,45,46,47,48,49], which have been intensively studied because the enzyme plays a key role in melanogenesis. TYRs, particularly the readily available TYR from *Agaricus bisporus*, have often been used as models for testing new inhibitors with potential application in medicine and cosmetics [46,48]. One review addressed the kinetics of TYR [50].

Recent studies have suggested new uses for Lacs and TYRs in the production of bioactive compounds and building blocks. Thus, they open pathways to sustainable organic syntheses. We believe that this important area of biocatalysis should be summarized and its perspectives discussed. First, this work summarizes the synthetic applications of Lacs based on the literature since approximately 2016, which was not included in previous reviews on a similar topic [51,52]. Second, the applications of tyrosinases are summarized analogously, based on the literature of the past decade. This is because a proper review of tyrosinase biocatalysts has been lacking during this period.

## 2. Laccase-Catalyzed Reactions

The ability of Lac to form radicals that result in various types of products such as oligo- and polymers has long been known. However, the scope of Lac products has greatly expanded since the last reviews [51,52]. In the following, we categorize the applications as homocoupling, heterocoupling, and other oxidation reactions.

### 2.1. Homocoupling

The improved biological activities or solubilities of oligomers of natural compounds [52] has prompted further research in this area. This improvement in the product properties has been explained by the multiplication of functional (hydroxyl) groups [53]. Recently, these types of products were prepared from several natural compounds such as piceid [54], lignols [55], rutin [56], caffeic acid [57,58], 4-hydroxychalcons [59], or isorhamnetin [59].

**Figure 2:**
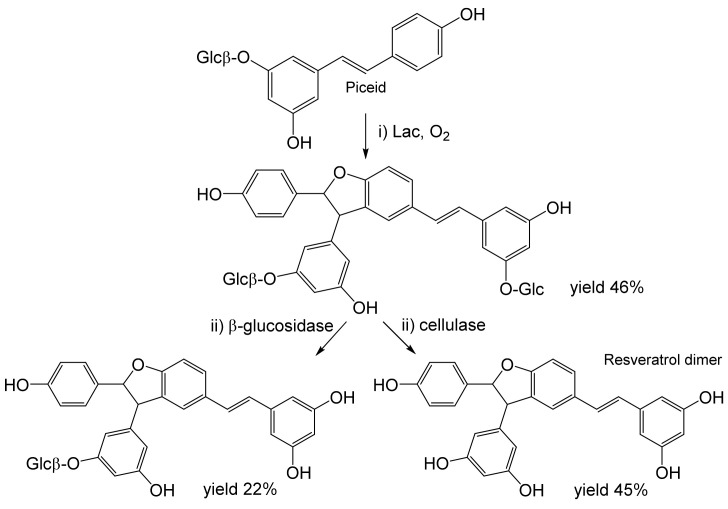
Dimerization of piceid (2.56 mmol) by laccase (18 mg; 86 U) from *Trametes versicolor* followed by deglucosylation of the dimer with β-glucosidase from almonds, or cellulase from *Trichoderma viride* [54]. Conditions: (i) acetate buffer (pH 5.0): MeOH, 71:29; 25 °C, 3.5 h (laccase); (ii) acetate buffer (pH 5.0): MeOH, 88:12, 25 °C (β-glucosidase), or acetate buffer (pH 5.0): MeOH, 93:7; 30 °C (cellulase).

Piceid is a β-glucopyranoside of resveratrol, a phytoalexin and well-known dietary supplement, although with uncertain bioactivity. Oxidation of piceid (≈18 mM) by Lac yielded a mixture of two dimeric glucosides, which were then partially or completely deglucosylated (Figure 2). The isolated yield of the resveratrol dimer (related to piceid) was ≈45%. In addition, its enantiomers, or the diastereomers of the glucosylated products, were separated by HPLC. Radical (2,2-diphenyl-1-picrylhydrazyl, DPPH) scavenging was weaker in the dimers than in piceid or resveratrol; nevertheless, the biological activities of the new dimers are of further interest [54].

**Figure 3:**
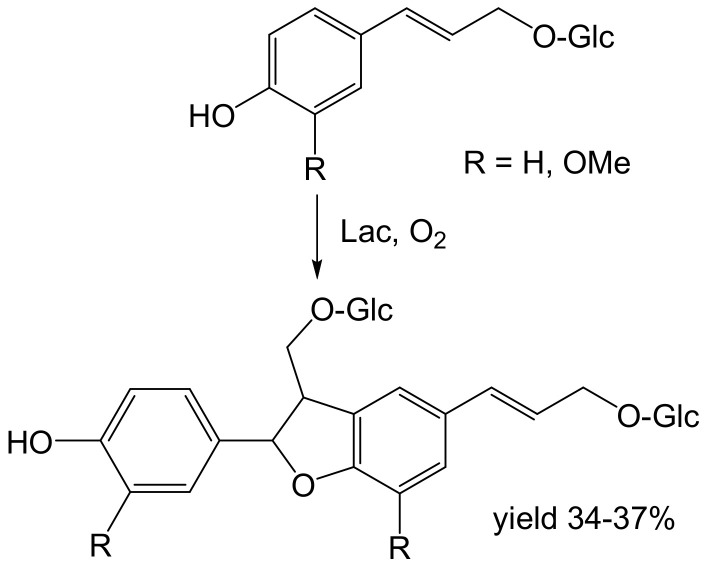
Dimerization of d-glucosides of the lignols coumaryl alcohol (R = H; 0.2 mmol) and coniferyl alcohol (R = OMe; 1.4 mmol) by laccase (0.32 and 2.35 U, respectively) from *Trametes versicolor*. l-Glucoside (0.89 mmol) or rutinoside (0.46 mmol) of coniferyl alcohol reacted analogously [55]. Conditions: acetate buffer (pH 5.0), 30 °C, 6–8 h.

Analogously, dimers were obtained from 50 mM glucosides of coumaryl and coniferyl alcohol (Figure 3). The bulkier diglycosylated (glucorhamnosyl) coniferyl alcohol was also a substrate [55]. The products of glycoside dimerization were prepared in acceptable (34–46%) yields in the tens to hundreds of mg scale [54,55]. The advantages of using glucosides as substrates are higher solubility and limitation of product diversity.

The low solubility of the substrate can also be addressed by medium engineering. Thus, the Lac-catalyzed reaction of the glycoside rutin was performed using an optimized (biphasic) system that consisted of ionic liquid (cholinium dihydrogen phosphate) and polyethylene glycol 600 [56]. The rutin oligomer, which is more water-soluble than rutin, was obtained with 95% conversion from a 3 g/L (≈5 mM) substrate. The conversion was still approximately 90% when using the catalyst two or three times. The reaction was also feasible at a substrate concentration of 10 g/L (≈16 mM), and the conversion was >93%.

**Figure 4:**
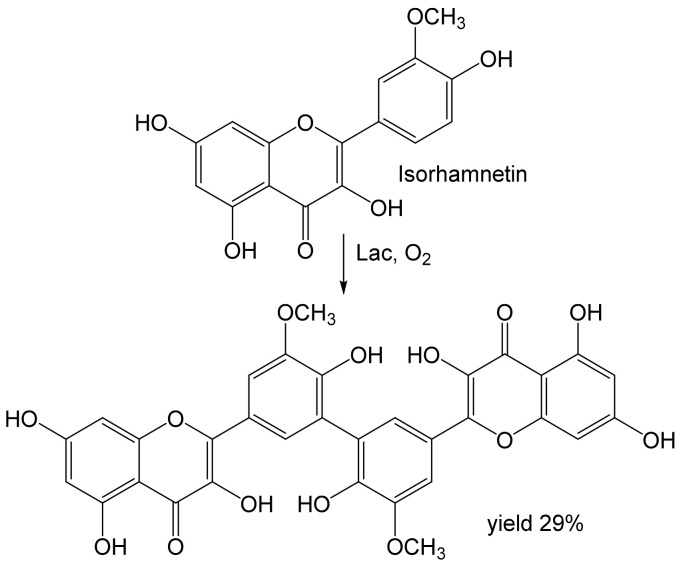
Dimerization of isorhamnetin (12.5 mM) by laccase from *Trametes pubescens* [53]. Conditions: acetate buffer (pH 5.0): EtOH, 1:1; 37 °C, 3 h.

Dimeric products were also obtained from flavonoid isorhamnetin [53] or caffeic acid [57,58]. The product of isorhamnetin (12.5 mM) was probably a C-C-linked dimer (Figure 4). It was obtained in a moderate yield (<30%) together with another (unidentified) product that was thought to contain a C-O-C bond. The DPPH scavenging activity of the first product and its ability to inhibit the growth of some bacteria (*Listeria*, *Staphylococcus*) was about twice that of the monomer.

**Figure 5:**
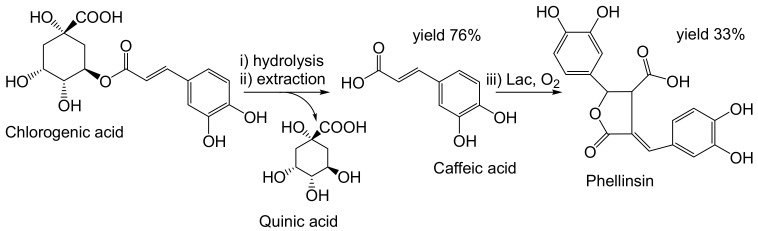
Production of phellinsin A, i.e., caffeic acid (CFA) dimer, from chlorogenic acid (CLA) [58]. Conditions: (i) 1.8 M NaOH, 0.1% ascorbic acid, 10 mM EDTA, r.t., 20 min; (ii) extraction of CLA with ethyl acetate (pH 3.0, saturation with NaCl); (iii) laccase (2 × 0.64 U/mL, phosphate buffer (pH 7.5): ethyl acetate, 1:4, 25 °C, 3 h. Optionally, CLA was obtained from potato peel. The yield of phellinsin A is related to CLA.

Dimerization of caffeic acid (CFA; 10 mM) to phellinsin A (Figure 5) increased radical scavenging activity by 50% and 80%, respectively, as determined by DPPH and Trolox assays [57], respectively. The catalyst was a heterologously-produced bacterial Lac resistant to detergents [57]. Optionally, potato peel (blended and lyophilized) was the source of chlorogenic acid (CLA; ≈0.5 mg/g potato peel powder). CLA (≈1 mM) upon alkaline hydrolysis yielded the substrate CFA and the by-product quinic acid [58]. The total yield of the process was ≈33%.

**Figure 6:**
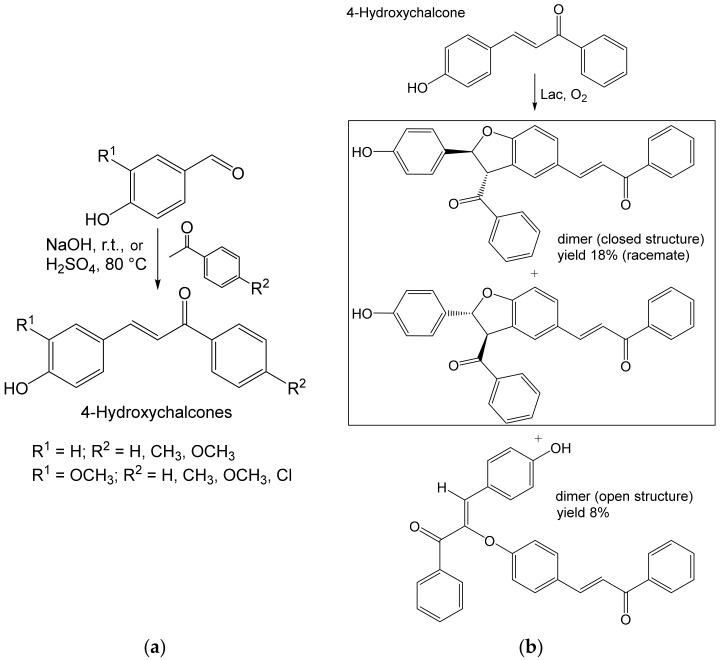
(**a**) Synthesis of 4-hydroxychalcones from acetophenone derivative and 4-hydroxybenzaldehyde or its derivative (2 mmol each) and (**b**) homocoupling of selected 4-hydroxychalcone (0.89 mmol) by laccase (20 mg, 90 U) from *Trametes versicolor* [59]. Conditions: (**a**) ≈0.6 M NaOH, EtOH; r.t., ≈20 h, or ≈0.5 M H_2_SO_4_, MeOH, 80 °C, 48 h; (**b**) acetate buffer (pH 4.5): acetone, 1:1; 27 °C, 6 h.

Two types of dimers were obtained by the action of Lac on 25 mM chalcones (1,3-diphenylprop-2-en-1-ones; plant compounds, radical scavengers) synthesized from benzaldehyde (or its analogs) and acetophenone analogs (Figure 6). One of the dimers (a racemate) contained the 2,3-dihydrobenzofuran scaffold occurring in many bioactive compounds. The mixtures of dimers obtained at the ten-mg scale (15–26% yields) were separated at the analytical or preparative scale [59].

**Figure 7:**
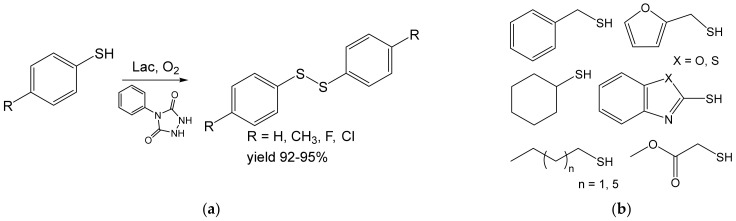
(**a**) Dimerization of thiophenols (1 mmol) by laccase from *Trametes versicolor* (46 mg, 40 U) [60]. (**b**) Other thiols react analogously. Conditions: phosphate buffer (pH 5.0), MeCN, 4:1, 4-phenyl urazole (10% mol., catalyst), r.t., 3–15 h.

A variety of thiols were shown to undergo an analogous reaction (Figure 7). The resulting disulfides are useful in (bio)chemistry as oxidants and protein stabilizers. They were prepared from 100 mM substrates 75–95% yields on a ten-mg scale. The reactions were performed with an innovative Lac-mediator (4-phenyl urazol) system [60].

### 2.2. Heterocoupling

**Figure 8:**
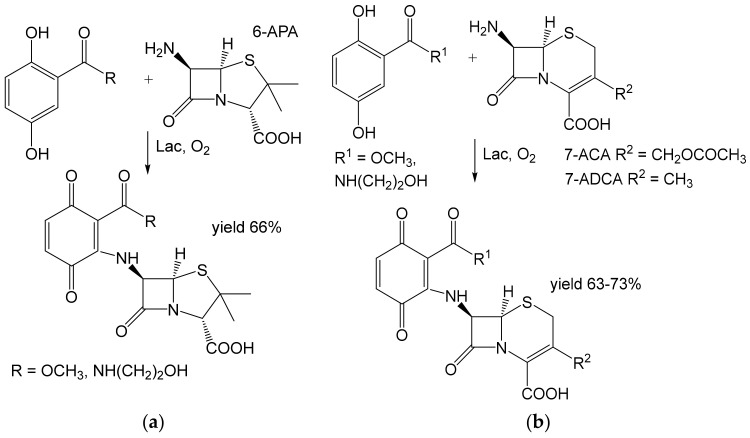
Heterocoupling of (**a**) 6-aminopenicillanic acid (6-APA) or (**b**) 7-aminocephalosporanic acid (7-ACA) and 7-aminodesacetoxycephalosporanic acid (7-ADCA) with 2,5-dihydroxybenzoic acid derivatives (1.2 mmol each) [61] by laccase (528 U) from *Trametes* sp. (Spezialenzyme, Wolfenbüttel, Germany). Conditions: acetate buffer (pH 5.6), r.t., 3 h.

**Figure 9:**
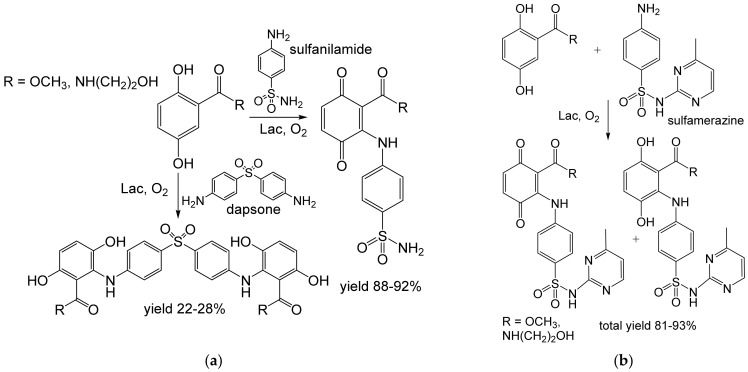
Heterocoupling of (**a**) sulfanilamide, dapsone, or (**b**) sulfamerazine with 2,5-dihydroxybenzoic acid derivatives (0.12 mmol each) [62] by laccase (53 U) from *Trametes* sp. (Spezialenzyme, Wolfenbüttel, Germany). Conditions: acetate buffer (pH 5.6), r.t., 6 h.

Recently, heterocoupling reactions were performed between β-lactam antibiotics [61], sulfanilamide, dapsone, or sulfamerazine [62] on one hand, and 2,5-dihydroxybenzoic acid derivatives on the other (Figure 8 and Figure 9). The latter were selected because a similar structure occurs in the antibacterial compound’s ganomycins. The products were obtained from 2 mM substrates in the ten-mg [62] to hundred-mg scale [61]. The yields were good to excellent (63–93%) except for the products from Dapson (22–28%). The hybrid antibiotics were active against some multi-drug resistant staphylococci [61,62].

**Figure 10:**
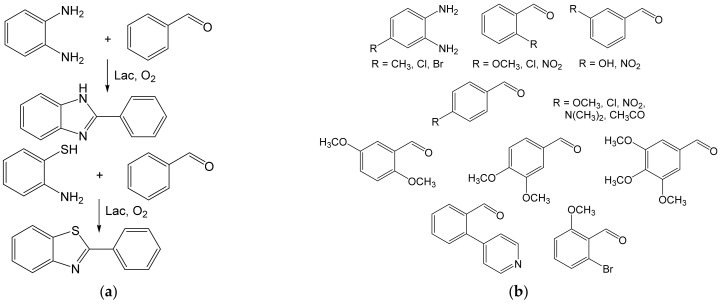
(**a**) Heterocoupling of benzaldehyde (20 mmol) and *o*-phenylenediamine (10 mmol) by laccase Novoprime Base 268 (0.105 g), or benzaldehyde (10 mmol) and *o*-aminothiophenol (15 mmol) by laccase Suberase^®^ (2 mL) [63]; (**b**) Some derivatives of benzaldehyde and *o*-phenylenediamine react analogously. Conditions: acetate buffer (pH 4.0): MeCN, 1:1; r.t., 2–24 h.

Heterocoupling was also used to prepare a variety of building blocks for organic synthesis. Thus, 2-arylbenzimidazoles and 2-arylbenzothiazoles were prepared from benzaldehyde and *o*-phenylenediamine, or benzaldehyde and *o*-aminothiophenol (Figure 10a) and a great variety of analogs (Figure 10b). The benzimidazols and benzothiazols were obtained in 56–94% and 48–88% yields, respectively, from ≈0.5–0.9 M benzaldehydes. Benzimidazole is a structural motif of omeprazole and is similar to drugs used to cure gastric disorders (hyperacidity, ulcers, etc.). The compounds of this type are also precursors of antibacterial, antiviral, and antitumor drugs; antiallergens; and antihypertensives [63].

**Figure 11:**
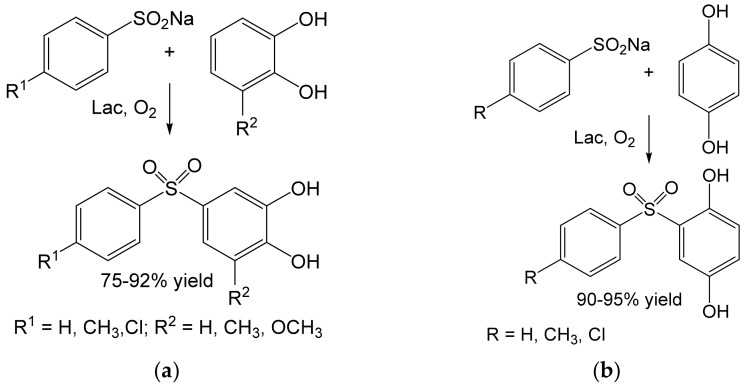
Heterocoupling of benzenediols and benzenesulfinates and (**a**) substituted catechols or (**b**) 1,4-benzenediol (1 mmol each) by laccase from *Trametes versicolor* (75.5 mg, 40 U) [64]. Conditions: phosphate buffer (pH 5.0); r.t., 18 h.

**Figure 12:**
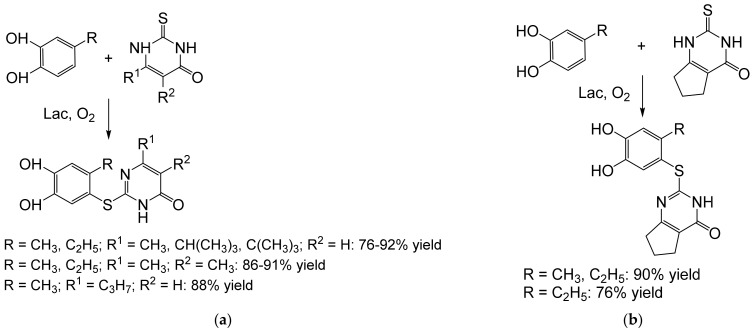
Hetero-coupling of benzenediols (0.58 mmol) and (**a**) 2,3-dihydro-2-thioxopyrimidin-4(1*H*)-ones (0.5 mmol) or (**b**) 2,3,6,7-tetrahydro-2-thioxo-1*H*-cyclopenta[*d*]pyrimidin-4(5*H*)-ones (0.5 mmol) by laccase (10 mg, 12 U) from *Agaricus bisporus* [65]. Conditions: phosphate buffer (pH 6.0), 10% EtOH; r.t., 12–20 h.

Heterocoupling of benzenediols (1,4-benzenediol or 3-substituted catechols) and benzenesulfinates led to diarylsulfones, known as structural motifs of the antibiotic dapsone, and various compounds with antifungal, antitumor, or antiviral activity. A set of these compounds were prepared in 75–95% yields on a hundred-mg scale [64] (Figure 11). Similarly, the corresponding catechol thioethers with cytostatic activity were prepared from 4-substituted catechols and pyrimidine analogs [65] (Figure 12). The concentrations of the diol substrates were ≈29 mM [65] and ≈67 mM [64]. Both types of hybrid products were prepared in good to excellent yields (75–95%) at the tens to hundred-mg scale.

### 2.3. Other Oxidation Reactions

Mild oxidation by Lac proved to be suitable for sensitive compounds such as 2-thiophenemethanol [66], propargyl alcohols [67], or secondary alcohols [68]. Thiophenemethanol (10 mM) was converted to 2-thiophenecarboxaldehyde by an immobilized Lac/TEMPO system on an analytical scale. Such thiocarbonyl compounds are important for the production of agricultural chemicals, pharmaceuticals, and dyes [66].

**Figure 13:**
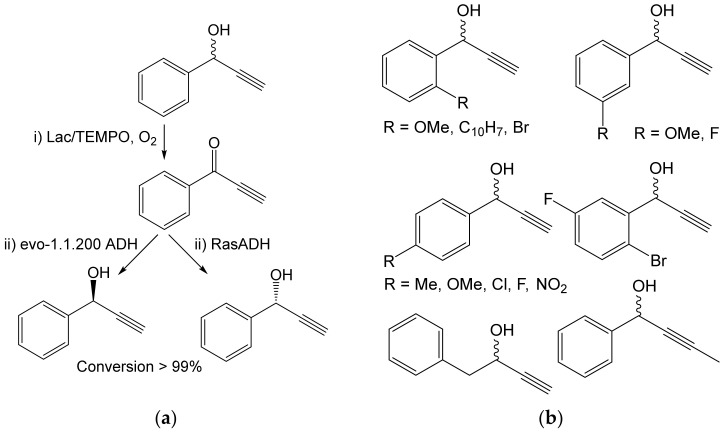
Oxidation of sensitive alcohols by laccase: (**a**) Oxidation of propargyl alcohols (0.05 mmol) by laccase from *Trametes versicolor* (10 mg, 5U) and subsequent reduction of the products by alcohol dehydrogenase (ADH) RasADH from *Ralstonia* sp. or evo-1.1.200 (Evoxx technologies, Düsseldorf, Germany) [67]. (**b**) Substrates undergoing the same reaction. Conditions: (i) Citrate buffer (pH 5.0), TEMPO (0.015 mmol), 10% *tert*-butyl methyl ether, 30 °C, 16 h; (ii) ADH, NAD(P)H, glucose dehydrogenase GCH-105 (Codexis Inc. Redwood, CA, USA), pH 7.5; 30 °C, 24 h.

An enzyme cascade consisting of Lac and alcohol dehydrogenase (ADH) was used for the production of enantiomerically pure propargyl alcohols, which are important building blocks. Deracemization of racemic alcohols (50 mM) was performed in one pot on a semipreparative scale. Depending on the source of ADH, both enantiomers were obtained with largely excellent conversions at up to >99% enantiomeric excess (Figure 13) [67].

**Figure 14:**
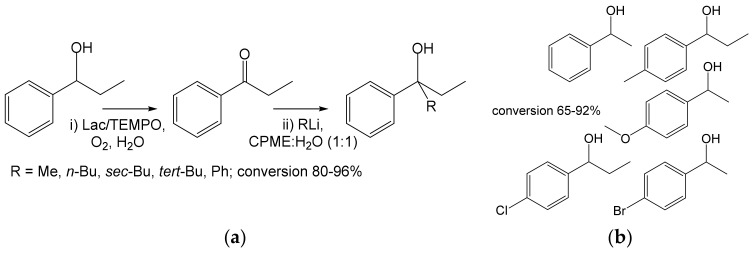
One-pot cascade from secondary to tertiary alcohols. (**a**) Conversion of 1-phenylpropan-1-ol (0.365 or 0.73 mmol) to tertiary alcohols using laccase from *Trametes versicolor* (140 U) and RLi reagents [68]. Conditions: (i) water, TEMPO (10 mol%), r.t., 24 h; (ii) water: cyclopentyl methyl ether (CPME), 1:1 (*v*/*v*), RLi (3 eq.), r.t., 3 s; (**b**) Secondary alcohols undergoing an analogous reaction with PhLi.

In addition, the synergy of Lac/TEMPO and organometallic compounds (RLi/RMgX) was used to transform a variety of secondary alcohols (0.73 M) into tertiary alcohols without isolating the intermediate ketones [68]. Under optimum conditions, the first step (oxidation by Lac) took place in an aqueous medium, while an organo-aqueous medium was suitable for the second (addition of RLi). The conversion was determined by GC and reached up to 80–91% (Figure 14).

### 2.4. Sources of Laccases

Most of the above reactions were performed with a Lac from *Trametes*. This enzyme is commercially available, e.g., from Sigma Aldrich and ASA Spezialenzyme. The latter company also produces several other Lacs from, e.g., *A. bisporus.* This enzyme was used in one of the above studies [65]. One of the Lacs from this company is a recombinantly produced enzyme recommended for organic synthesis (http://asa-enzyme.com/products/special-enzymes; accessed on 27 February 2022).

In addition, two Lacs from *Myceliophthora thermophila* (Suberase^®^; Denilite^®^ II Base) were prepared by Novozymes, together with the Lac Novoprime Base 268. The last was evaluated as the best one in terms of chemoselectivity [63].

Commercial enzymes are not inexpensive. They have been used for tens to hundreds of mg-scale preparative reactions (see above), but scale-up can be costly. Some researchers have produced these enzymes themselves, such as laccase from *Trametes pubescens*. This extracellular enzyme was precipitated from the cultivation medium with ammonium sulfate [53]. Bacterial Lacs have rarely been used. An exception was a “small Lac“ (SLac) from *Streptomyces coelicolor.* The enzyme was produced in *E. coli* and purified [57,58].

There exist a number of other Lacs that have been studied for other applications but may also be useful for organic synthesis. For example, the cotA laccase from *Bacillus subtilis* is suitable for alkaline conditions. This was recently demonstrated by its use in the modification of Kraft lignins. The enzyme was produced in *E. coli* and purified in one step [69].

Fungi have been known as sources of Lacs for decades, but their potential has not yet been fully exploited. Namely, the understanding of Lac production in wild-type producers needs to be improved. *Ganoderma lucidum*, for example, contains several *lac* genes, whose transcription depends on pH. In the past, the focus has been on extracellular Lacs, but some fungi, including *G. lucidum*, may also produce intracellular Lacs. The study of these enzymes may open new perspectives in this research [70]. 

The heterologous expression systems suitable for Lacs were summarized in [18]. These include standard bacterial and yeast hosts but also insect cells. The order Agaricales has been less studied for Lacs than the order Polyporales [71]. A recently used system was based on *Saccharomyces cerevisiae* and the gene originated from the fungus *Agrocybe pediades* (Agaricales). A double mutant of this Lac is a promising catalyst in terms of redox potential and pH range (shifted to neutral values) [71].

Another way to improve Lacs is their immobilization. The general methodology and the uses of immobilized Lacs have been summarized previously [19]. Recent advances in this research include the use of cross-linked magnetic nanoparticles (NPs) [6] or hybrid Lac-copper phosphate and Lac-zinc phosphate catalysts [14,72]. These methods resulted in increased stability of the enzyme and allowed recycling of the catalyst. For example, the copper NPs (nanoflowers) were recycled ten times and retained over 92% activity [14]. The catalysts have been tested for the decolorization of synthetic dyes [14] or bisphenol A degradation [6], but other applications are also possible.

## 3. Tyrosinase-Catalyzed Reactions

### 3.1. Ortho-Hydroxylation of Phenols

The main application of TYR is the production of valuable benzene-1,2-diols (catechols) from the cheaper and readily available phenols. Catechols are often biologically active due to their ability to chelate metal ions and scavenge radicals. The synthesis of catechols consists of two coupled reactions—oxidation of phenols to *ortho*-quinones and the chemical reduction of the quinones. This one-pot pathway was established in 2001 for the production of hydroxytyrosol, a naturally occurring compound in olives [73]. Later this method and its variants were used to prepare a large number of catechols of diverse structures (see below).

**Figure 15:**
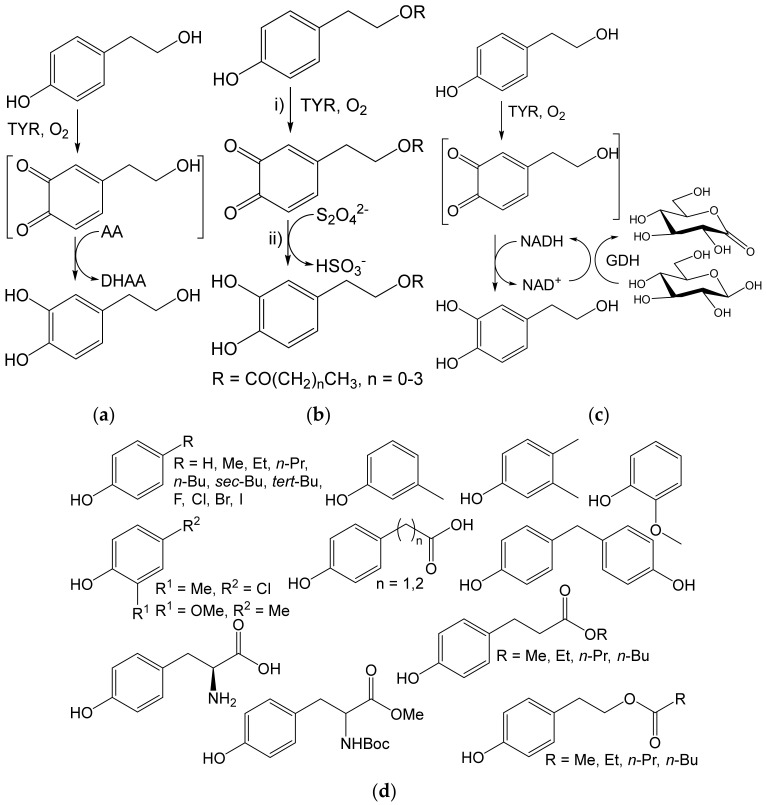
*ortho-*Hydroxylation of tyrosol by tyrosinase. (**a**) One-pot reaction of tyrosol (16 mM) with tyrosinase (30 μg/mL) from *Agaricus bisporus* (Sigma) [73]. Conditions: phosphate buffer (pH 6.5), 30 mM ascorbic acid (AA); DHAA, dehydroascorbic acid. (**b**) Cascade reaction of tyrosol esters (0.05 mmol) using immobilized tyrosinase (240 U) from *A. bisporus*. Conditions: (i) Buffer (pH 7.0): CH_2_Cl_2_, 1:10, 25 °C, 24 h; (ii) Na_2_S_2_O_4_ (in THF:H_2_O, 1:1) [75]. (**c**) One-pot reaction of tyrosol (1 mM, 0.35 mmol) with immobilized tyrosinase (0.7 g) from *Bacillus megaterium.* Conditions: Buffer (pH 7.5), 7 g immobilized glucose dehydrogenase (GDH), 0.4 mM Cu^2+^, 100 mM d-glucose, 5 mM NAD^+^; 25 °C, ≈2 h [76]. (**d**) Phenol compounds undergoing analogous reactions according to (**a**) and/or (**b**) [74,75,77,78,79,80,81,82].

The use of TYR for the preparation of hydroxytyrosol from tyrosol (16 mM) on an analytical scale was based on the combination of TYR as an oxidizing agent and ascorbic acid (30 mM) as a reducing agent [73]. The intermediate quinone is reduced to hydroxytyrosol (Figure 15a). The same principle has been used to convert 50 mM tyrosol and other phenols to the corresponding catechols on a preparative scale. The use of the soluble or immobilized TYR enabled 77–85% yields of hydroxytyrosol and almost quantitative yields of some other catechols [74]. Hydroxytyrosol is known as a radical scavenger and generally as a health-promoting compound. Hydroxytyrosol esters were also prepared from 50 mM tyrosol esters using sodium dithionite as a reducing agent, which was added after the completion of the phenol conversion (Figure 15b) [75]. In this case, the aqueous medium was replaced by an organo-aqueous medium (dichloromethane with ≈10% phosphate buffer). The enzyme had to be immobilized to work in this medium [75]. Recently, a new variant of this method was used to produce hydroxytyrosol. The reducing agent was NADH recycled using glucose dehydrogenase (GDH) (Figure 15c). This hydroxytyrosol production is competitive with, e.g., microbial hydrolysis of oleuropein or the use of cell factories (in terms of product yield and concentrations) [76].

Both the one-pot and cascade methods (Figure 15a,b) also proved to be useful for the *ortho-*hydroxylation of other phenol compounds (Figure 15d). These were, e.g., 4-hydroxyphenylacetic acid [80], 4-hydroxyphenylpropionic acid (phloretic acid) [80] and its esters [75], l-tyrosine [83,84] and its derivatives [77,78], 4-alkylphenols [74,80,81], 4-halophenols [74,80,82], other substituted phenols, and bisphenol A [74,80]. The targeted catechols were produced from 10–50 mM phenols in the mg to tens of mg range. The isolated yields were largely excellent and any byproducts (dimers, trihydroxyphenols) were minor [80]. Their production depended on the medium (presence of an organic solvent) and the enzyme form (soluble vs. immobilized). The organic solvent (dichloromethane) generally suppressed their formation. The cascade method was also used for the preparation of peptides, in which the tyrosine residue was converted to 3,4-dihydroxy-L-phenylalanine (DOPA) [80].

DOPA has been known for decades for its effect in the treatment of parkinsonism. The peptides from DOPA are expected to have a better effect in this sense. These peptides could also be useful in the treatment of atherosclerosis and in cosmetics [78]. The esters of hydroxytyrosol and 3,4-dihydroxyphenylpropionic (dihydrocaffeic) acid with lipophilic side chains attracted attention as potential antiviral agents [75,79]. Some alkylphenols with a short alkyl chain (e.g., methyl, ethyl) are promising as agents against oxidative stress [85]. This activity remains to be investigated in similar catechols with longer side chains.

### 3.2. Sources of Tyrosinases

The most commonly used TYR is derived from the common button mushroom, *Agaricus bisporus*, and is commercially available. Its immobilization was especially beneficial when the reaction was carried out in a largely organic medium. The catalyst was immobilized on Eupergit, and this preparation was then modified with a layer-by-layer (LbL) coating, i.e., it was covered with layers of electrolytes with opposite charges. This catalyst was recycled five times with 75% activity after the last run [74]. A variant of this method consisted of attaching TYR to carbon nanotubes followed by LbL coating [75]. It exhibited excellent stability during recycling; the yield of caffeic acid decreased from 98% to 91% in the sixth run. In another study, immobilization of a commercial TYR on a polyamide membrane enabled continuous production of DOPA in a laboratory-scale bioreactor [83].

The *A. bisporus* TYR can also be obtained in the laboratory from the fruiting bodies of the fungus. The methods for its production have already been discussed in relation to its potential for biodegradation [22]. This enzyme (partially purified by ammonium sulfate precipitation) proved useful for the synthesis of DOPA and DOPA peptides [78] or *n*-alkylcatechols [81]. It was also immobilized on Eupergit and coated by the LbL method [78], much like the commercial enzyme (see above). Determination of both commercial and crude TYR of *A. bisporus* indicated that the former has more than five times higher specific activity for L-tyrosine [80] or DOPA [81]. In addition, this TYR (in the form of its isoenzymes) and other fungal TYRs have been recombinantly produced [86,87,88,89,90,91,92]. However, this source is still underutilized in biocatalysis.

Bacterial TYRs were used for the production of halocatechols (TYR from *Ralstonia solanacearum*) [82] and hydroxytyrosol (TYR from *Bacillus megaterium*) [76]. Both enzymes were overproduced in *E. coli* and purified. In addition, artificial variants of the former enzyme were generated by single-point mutation and were found to be superior in terms of its kinetics for halophenols. The TYR of *B. megaterium* was used in a sol-gel immobilized form. It proved suitable for both continuous and repeated use (with an almost full conversion of tyrosol in the eighth run) [76].

There are also other TYRs that can be useful in organic synthesis. For example, the TYRs of *Pholiota microspora* [87,88], *Streptomyces antibioticus* [89] or *Polyporus arcularius* [90] were produced recombinantly. Although *E. coli* can be used, expression in this host results in an inactive pro-enzyme, which must be activated by proteolysis [87,88,90]. In contrast, the enzyme is processed in *Aspergillus niger* [91] or *Komagataella phaffii* (formerly *Pichia pastoris*) [92]. On the other hand, the presence of an active enzyme may be detrimental to the host, because the enzyme is capable of oxidizing Tyr residues. The TYRs of *S. antibioticus*, *P. aucularius*, and *A. bisporus* have been examined as models for inhibitor studies. However, they have also been shown to transform some (potentially bioactive) derivatives of aurons and may be useful for their modifications [89,90].

## 4. Major Challenges and Prospects

The above research addressed the major challenges of using Lac and TYR in organic synthesis. These are to prepare effective catalysts and to design selective reactions with high product yields. Some work also addressed the structure–activity relationships (SAR) of bioactive products.

A few Lacs and TYRs are readily available from commercial suppliers. These have been sufficient for small-scale syntheses and SAR studies. The immobilization of these enzymes, such as the Lac from *T. toxicodendron* [6,72] and *T. versicolor* [14], or the TYR from *A. bisporus* [75,78,79,80], was promising. The obtained catalysts showed improved properties in terms of thermal and pH stability resistance to solvents/detergents, shelf-life, and/or recyclability.

Some Lacs and TYRs can be prepared by simple methods for direct use. Lacs are available from the culture fluid of fungal cultures and can be used without purification [93]. However, the cultivation of fungi may be beyond the routine work of an organic chemist. In contrast, TYR can be prepared by simple extraction of fruiting bodies of the common button mushroom and, optionally, by ammonium sulfate precipitation [81].

The discovery of new Lacs and TYRs, including the hitherto poorly studied bacterial enzymes [57,58,76,82] suggests that the scope of these catalysts may be extended. Recombinant production of Lac and TYRs for organic synthesis is still poorly explored. The availability of commercial services such as gene synthesis and overexpression make this option accessible also to non-biologists.

Developing processes selected for upscaling requires overcoming additional challenges. Minimizing catalyst costs is one of the most important. Commercial enzymes are relatively expensive, although they are likely to be available in bulk quantities at reduced prices. In this context, the development of the stable immobilized catalysts mentioned above is an important advance that can be applied to other Lacs or TYRs. The combination of an overproduced thermostable enzyme with a suitable immobilization may be the best way to obtain low-cost catalysts.

The space-time yield (g product/L/h) must be optimized to make the process viable. This factor depends on the concentration and conversion of the substrate. Most of the above reactions were demonstrated with substrate concentrations of ≤50 mM. Those performed with higher substrate concentrations (0.1 M thiols [60], ≈0.5 M *o*-phenylenediamine, ≈0.7 M *o*-aminothiophenol [63], or ≈0.7 M secondary alcohol [68]) have been the exception. The space-time yield was calculated to be 0.16 g/L/h and 0.69 g/L/h for 50 mM and 10 mM substrate, respectively, for hydrotyrosol production. Thus, it was relatively low, especially at the higher substrate concentrations. This was partly due to the inhibition of the enzyme by the product. Therefore, product removal by adsorption, for example, was suggested [76].

The performance of other reactions used in the process must be taken into account, and reaction conditions must be adjusted, especially for one-pot reactions. Many of the reactions demonstrated were two- or multi-step. Virtually all phenol-catechol reactions combined oxidation by TYR with chemical reduction by ascorbic acid [73,74,81], dithionite [75,78,80] or NADH. The recycling of NADH is necessary for a viable process, and it has been achieved using an additional enzyme—GDH. Moreover, both TYR and GDH were immobilized to allow recycling [76].

The multi- and chemoenzymatic reactions were also used to increase selectivity (dimerization of glucoside followed by deglycosylation [54,55]), to enable deracemization (one-pot reaction catalyzed by Lac and ADH [67]) or to utilize a cheap substrate (dimerization of CFA obtained from waste material [58]). We assume that these processes are particularly promising, as they maximize and valorize the synthetic potential of Lacs and TYRs.

## 5. Conclusions

The use of Lacs and TYRs opens up simple solutions for the synthesis of valuable (hetero) aromatic compounds. The alternative chemical routes are often complex and involve protection and deprotection steps, strong acids or bases, expensive or toxic oxidants, or high temperatures. Several Lacs and the TYR from *A. bisporus* are available from commercial companies. This has allowed organic chemists to directly develop new “green” syntheses that yielded compounds promising as dietary supplements, pharmaceuticals, and building blocks for fine chemicals. Known bioactive compounds were modified by dimerization, oligomerization, or heterocoupling, and new ones were proposed. The enzymes have been very useful for the production of small amounts of the compounds for SAR studies. The biological tests of these products allowed the identification of structural motifs important for biological activities in some cases. The applications of the enzymes in the fine-chemical industry are plausible but it is essential to solve demanding tasks connected to scale-up.

## Data Availability

Not applicable.
